# Virulence is associated with daily rhythms in the within‐host replication of the malaria parasite *Plasmodium chabaudi*

**DOI:** 10.1111/eva.13696

**Published:** 2024-05-08

**Authors:** Alíz T. Y. Owolabi, Petra Schneider, Sarah E. Reece

**Affiliations:** ^1^ School of Biological Sciences, Institute of Ecology and Evolution University of Edinburgh Edinburgh UK

**Keywords:** asexual replication, circadian misalignment, circadian rhythm, host–parasite interactions, infection severity, intraerythrocytic developmental cycle

## Abstract

Most malaria (*Plasmodium* spp.) parasite species undergo asexual replication synchronously within the red blood cells of their vertebrate host. Rhythmicity in this intraerythrocytic developmental cycle (IDC) enables parasites to maximise exploitation of the host and align transmission activities with the time of day that mosquito vectors blood feed. The IDC is also responsible for the major pathologies associated with malaria, and plasticity in the parasite's rhythm can confer tolerance to antimalarial drugs. Both the severity of infection (virulence) and synchrony of the IDC vary across species and between genotypes of *Plasmodium*; however, this variation is poorly understood. The theory predicts that virulence and IDC synchrony are negatively correlated, and we tested this hypothesis using two closely related genotypes of the rodent malaria model *Plasmodium chabaudi* that differ markedly in virulence. We also test the predictions that, in response to perturbations to the timing (phase) of the IDC schedule relative to the phase of host rhythms (misalignment), the virulent parasite genotype recovers the correct phase relationship faster, incurs less fitness losses and so hosts benefit less from misalignment when infected with a virulent genotype. Our predictions are partially supported by results suggesting that the virulent parasite genotype is less synchronous in some circumstances and recovers faster from misalignment. While hosts were less anaemic when infected by misaligned parasites, the extent of this benefit did not depend on parasite virulence. Overall, our results suggest that interventions to perturb the alignment between the IDC schedule, and host rhythms and increase synchrony between parasites within each IDC, could alleviate disease symptoms. However, virulent parasites, which are better at withstanding conventional antimalarial treatment, would also be intrinsically better able to tolerate such interventions.

## 1 INTRODUCTION

Circadian rhythms have evolved to allow organisms to anticipate environmental cycles that have 24‐h periodicity (Panda et al., 2002; Patke et al., 2020). The daily rhythms in behaviour and physiology exhibited by hosts impose a rhythmic environment on their parasites, and so can mediate host–parasite interactions (Carvalho Cabral et al., 2019; Hunter et al., 2022; Prior et al., 2020; Westwood et al., 2019). For example, the time of day when infection occurs can determine the life‐or‐death outcomes of infection for taxonomically diverse hosts, including *Arabidopsis*, *Drosophila* and mice (Halberg et al., 1960; Heipertz et al., 2018; Hevia et al., 2015; Sengupta et al., 2019; Stone et al., 2012). Time‐of‐day effects of infection are in part explained by circadian clock control of the host's immune responses; for example, in mammals, leukocyte abundance, phagocytic activity and cytokine release all oscillate over the circadian cycle (Scheiermann et al., 2013). While such rhythms allow hosts to prepare effective defences against invading pathogens and parasites, host rhythms can also provide infectious agents with opportunities. For instance, invasion and replication of *Leishmania* parasites are facilitated by the accumulation of their host cells (macrophages) in the blood during the host's rest phase (Kiessling et al., 2017). The filarial nematodes *Brugia malayi* and *Wuchereria bancrofti* sequester in tissues in a rhythmic manner, which may be to ensure they are only exposed to dangers from immune defences in the peripheral circulation at the time of day when their mosquito vectors are seeking blood (Hawking, 1967; Pichon & Treuil, 2004). Parasites are not merely passive bystanders to environmental oscillations, and consequently, some have evolved autonomy over their own (endogenous) rhythms. For example, the fungus *Botrytis cinerea* uses a canonical transcription–translation feedback loop (TTFL) oscillator to schedule rhythms in factors used for host exploitation (Hevia et al., 2015). African trypanosomes and malaria (*Plasmodium*) parasites also fulfil some of the criteria for endogenous oscillators, but their molecular underpinnings remain mysterious (O'Donnell et al., 2021; Prior et al., 2021; Rijo‐Ferreira et al., 2017; Smith et al., 2020; Subudhi et al., 2020).

While rhythmicity in parasite activities is assumed to be advantageous, a population of parasites within a host all carrying out the same behaviour at exactly the same time may be intrinsically costly (Greischar et al., 2014). For example, malaria parasites replicate asexually in the red blood cells (RBCs) of their mammalian hosts, in a process termed the intraerythrocytic developmental cycle (IDC). In every IDC, a small fraction of asexual stages commit to becoming sexual stages (gametocytes), which are responsible for transmission to a mosquito vector and, ultimately, onward transmission to a vertebrate host. The IDC in most malaria species involves parasites progressing synchronously and sequentially through three main developmental stages (rings, trophozoites and schizonts). Broadly, the role of rings is to remodel the RBC; trophozoites feed and grow; and schizonts are responsible for producing progeny (merozoites), which are released at the end of the IDC (schizogony) (Tuteja, 2007). Synchronous schizogony, which causes recurring fevers at 24‐, 48‐ or 72‐h intervals, is a hallmark of many *Plasmodium* species (Gautret et al., 1995; Hawking et al., 1968; Mideo et al., 2013; O'Donnell et al., 2011; Simpson et al., 2002). Schizogony in the rodent malaria model *P. chabaudi* follows a 24‐h rhythm and is timed to align with host feeding–fasting rhythms (Hirako et al., 2018; O'Donnell et al., 2020, 2021; Prior et al., 2018). Specifically, IDC completion occurs during the feeding window, i.e. the 12‐h dark period for nocturnal mouse hosts, and this temporal coordination between host and parasite is likely explained by when rhythmic host nutrients – including the food‐derived essential amino acid isoleucine – peak in the blood (Prior et al., 2021). Thus, the timing (phase) of the IDC is thought to facilitate resource acquisition, but closely genetically related parasites that are perfectly synchronised (i.e. a high amplitude rhythm) may inadvertently compete for access to nutrients. In addition to “crowding” causing competition for time‐limited nutrients, highly synchronous bursting may cause merozoite progeny to hinder each other's access to red blood cells (RBCs; Greischar et al., 2014).

The costs of synchrony are likely to be greater when parasites are at high density, when hosts have reduced appetite due to infection symptoms and/or when parasites exist in genetically mixed infections in which they must compete with rival, unrelated genotypes. Malaria parasite genotypes vary in within‐host replication rates and densities achieved in the blood in manners related to their virulence (defined here as the degree of harm caused to the host; Bell et al., 2006). For malaria parasites, replication rate and virulence are related to immunogenicity (through host immunopathology), sequestration and cytoadherence to the microvasculature and clumping (rosetting) (Cockburn et al., 2004; De Niz et al., 2016; Long & Graham, 2011). Due to the higher densities of virulent genotypes, synchrony is more likely to cause inadvertent resource competition amongst closely genetically related parasites. Indeed, observational data (Ciuffreda et al., 2020; Dobaño et al., 2007; Simpson et al., 2002; Touré‐Ndouo et al., 2009) and mathematical modelling (Greischar et al., 2014) predict that the synchrony of the IDC is associated with virulence. For example, synchronous *P. falciparum* infections are sometimes associated with low parasitaemia (Ciuffreda et al., 2020) and the loss of synchrony during *P. chabaudi* infections coincides with high parasite density and RBC limitation (O'Donnell et al., 2021). These observations could be explained by synchrony limiting virulence. Put another way, avirulent parasites may garner the benefits of timing the IDC to align with host rhythms, with fewer costs of a trade‐off imposed by synchrony.

We test whether the IDC rhythm is linked to virulence using two very closely related (isogenic) genotypes of the rodent malaria *P. chabaudi* that differ substantially in virulence (Mackinnon & Read, 1999). Specifically, we hypothesise that the more virulent genotype, which reaches higher densities and depletes more host resources (including RBCs), should have a less tightly synchronised IDC. Furthermore, following perturbation of the IDC schedule relative to the rhythm of the host (misalignment), parasites readily reschedule to regain alignment with host rhythms within 5–7 IDCs (Gautret et al., 1995; O'Donnell et al., 2021; Subudhi et al., 2020). Plasticity in the IDC rhythm involves shortening the duration of each IDC by approximately 2 h (O'Donnell et al., 2021), potentially by downregulating the expression of Serpentine Receptor 10 (SR‐10) (Subudhi et al., 2020). Virulent genotypes are better able to cope with adverse within‐host conditions, including antimalarial drug treatment and competition in mixed‐genotype infections (Bell et al., 2006; De Roode et al., 2005; Schneider et al., 2008, 2012). Connecting these three observations leads us to hypothesise that the more virulent genotype suffers less from being misaligned and realigns to the phase of host rhythms faster. Our predictions are partially supported by data suggesting that the more virulent genotype is less synchronous, does not experience an increase in synchrony during misalignment and recovers the correct IDC rhythm sooner. However, we do not find that the impact of misalignment on overall asexual or gametocyte densities depends on virulence, and while disease severity is reduced by misalignment, this is not in a virulence‐dependent manner. Given that the IDC is responsible for the severity of malaria infections and fuels the production of transmission stages, our data suggest that disrupting the timing but not synchrony (or increasing synchrony) of the IDC is a novel approach to treat infections and reduce transmission (Prior et al., 2020).

## 2 METHODS

### 2.1 Hosts and parasites

We used the synchronously replicating rodent malaria parasite species *Plasmodium chabaudi chabaudi*, which was originally isolated from the shining thicket rat *Grammomys poensis* (previously called *Thamnomys rutilans*) (Landau, 1965), though rodent malarias also use *Mus musculus* as a natural host (Boundenga et al., 2019). Experimental hosts were 8–13‐week‐old female C57BL/6Jcrl mice (bred in house), with ad libitum access to food and to drinking water supplemented with 0.05% para‐aminobenzoic acid to support parasite growth (Jacobs, 1964). Data were collected from experimental hosts infected with parasites raised in donor mice (11‐week‐old female MF1). Experimental mice were housed in a standard 12‐h light:12‐h dark photoschedule with lights on [Zeitgeber Time 0; ZT0] at 10 am GMT and lights off at 10 pm GMT [ZT12]. Donor mice were housed in either the standard or reversed (lights on at 10 pm GMT [ZT0], lights off at 10 am GMT [ZT12]) photoschedules. All donor and experimental hosts were entrained into their photoschedule for 3.5 weeks prior to infection (O'Donnell et al., 2021; Schneider, Rund, et al., 2018). All procedures occurred in accordance with the UK Home Office regulations (Animals Scientific Procedures Act 1986; SI 2012/3039; project licence number 70/8546) and were approved by The University of Edinburgh.

Experimental hosts received 1 × 10^7^ red blood cells (RBCs) infected with ring stage parasites and diluted in citrate saline (0.85% w/v NaCl, 1.5% w/v trisodium citrate dihydrate), belonging to either the relatively avirulent parental clone CW839 (referred to as CW‐0) or its virulent progeny clone CW840 (referred to as CW‐VIR). Prior to our study, CW‐VIR parasites were produced by serially passaging parasites from the CW‐0 parent from the mice within each selection cycle that experienced the most severe weight loss. After 11 cycles of selection, CW‐VIR achieved a 2.61‐fold higher peak parasitaemia and caused 1.23‐fold lower mean RBC density and 1.68‐fold more mean weight loss than CW‐0 (Mackinnon & Read, 1999). Using avirulent and virulent parasites of the same genetic background in our experiment minimises the influence of other phenotypes that exhibit genetic variation and that could confound the influence of virulence on IDC characteristics (Birget et al., 2019; Pollitt et al., 2011).

### 2.2 Experimental design

**FIGURE 1:**
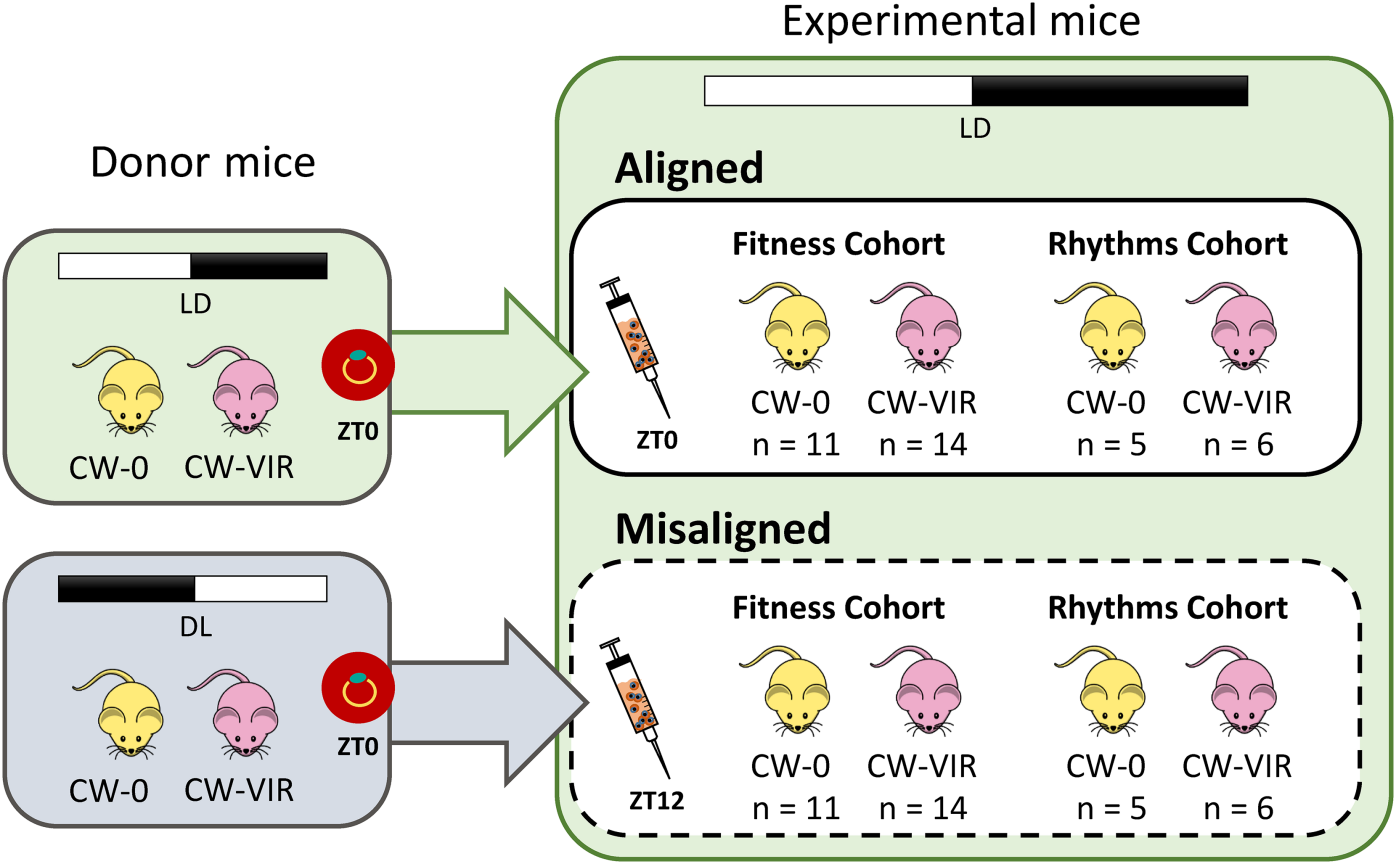
Experimental design. We infected experimental mice, housed in the standard photoschedule (green, LD), with 10^7^ ring‐stage parasites of either the relatively avirulent *Plasmodium chabaudi* CW‐0 (yellow) or the more virulent CW‐VIR (pink) genotype. For each genotype, parasites originated from donor mice housed in the standard photoschedule (green, LD) for ‘aligned’ groups or housed in the reversed photoschedule (grey, DL) for ‘misaligned’ groups where the alignment of the intraerythrocytic developmental cycle (IDC) to the rhythms of experimental hosts was perturbed. We replicated these four treatment groups in two cohorts, using the‘Fitness cohort’ to assess parasite performance and disease severity and the ‘Rhythms cohort’ to characterise the IDC schedule.

We designed a cross‐factored experiment (Figure 1) in which each genotype (CW‐0 and CW‐VIR) was introduced into experimental hosts whose rhythms followed the same timing as the IDC (‘aligned’) or had rhythms 12 h out of synch with the IDC (‘misaligned’). Establishing aligned infections involved infecting experimental hosts at ZT0 with ring stage parasites from donor mice in the same photoschedule, whereas we established misaligned infections 12 h later by infecting experimental hosts at their ZT12 with ring stage parasites from donors in the opposite (reversed) photoschedule (so at ZT0) to create an instantaneous 12‐h shift in the phase (timing) of host rhythms experienced by inoculated parasites. This design also standardised the IDC stage used to initiate infections and enabled aligned and misaligned parasites to be sampled at the same time points within their host's circadian cycle. To initiate infections, blood from CW‐0 or CW‐VIR donors within each photoschedule was pooled to minimise donor effects, and we randomly allocated experimental hosts to the four treatment groups and cohorts.

We initiated each of the four treatment groups in two cohorts of mice, termed the ‘Fitness’ and ‘Rhythms’ cohorts. Mice in the Fitness cohort (CW‐0: *n* = 11/group, CW‐VIR: *n* = 14/group, Figure 1) received parasites by intraperitoneal injection, and we followed their infections daily to measure the performance of parasites and disease severity. Mice in the Rhythms cohort (CW‐0: *n* = 5/group, CW‐VIR: *n* = 6/group, Figure 1) received parasites by intravenous injection, and we undertook 3‐hourly sampling from 42 to 75 h PI for mice with aligned infections and 30–63 h PI for mice with misaligned infections to estimate parameters describing the IDC rhythm. We used different cohorts for these purposes because the repeated sampling necessary to document IDC rhythms eventually generates anaemia and disrupts host rhythms, confounding the assessment of parasite performance and virulence (Prior et al., 2019). A consequence of this constraint is that the accuracy of period estimates (rhythm duration) may be affected by sampling not covering multiple IDCs. Further, intravenous infection ensured parasites in the Rhythms cohort replicated to high enough densities to be quantifiable before those in misaligned infections had rescheduled, whereas intraperitoneal injection slows infection progression, avoiding the risk of mice in the Fitness cohort prematurely succumbing to infections.

### 2.3 Sampling and data collection

We sampled the *n* = 50 infections in the Fitness cohort daily at ZT0 from days 3 to 16 post‐infection (PI) to quantify the densities of asexual stages and sexual transmission stages (gametocytes) and to track disease severity via weight (using a Kern & Sohn veterinary scale) and RBC density (measured by flow cytometry using a Beckman–Coulter Counter) by adding 2 μL of blood to 80 mL of Beckman Coulter™ ISOTON™ II Diluent (Fisher Scientific™) (Ferguson et al., 2003). We confirmed experimental mice in the Fitness cohort did not differ in their health parameters (weight and RBC density) between treatment groups 1 day before experimental infections began (Table S1). To determine total parasite density, we extracted DNA from 5 μL of whole blood using a semi‐automatic Kingfisher Flex Magnetic Particle Processor and MagMAX™‐96 DNA Multi‐Sample Kit (Thermo Fisher Scientific) with slight modifications from the standard protocol 4413021DWblood (Schneider et al., 2019a; Schneider, Greischar, et al., 2018), followed by amplification of the SOP1 gene (PCHAS_0620900, previously named PC302249.00.0 or CG1, DNA present in both asexual parasites and gametocytes) by qPCR (Wargo et al., 2007). To quantify gametocytes, we extracted RNA from 10 μL of whole blood using the MagMAX™‐96 Total RNA Isolation Kit on the Kingfisher machine (Birget et al., 2017; Schneider et al., 2019b), followed by reverse transcriptase qPCR targeting the SOP1 gene, which is expressed only in gametocytes (RNA present only in gametocytes; Wargo et al., 2007).

We took blood smears from the *n* = 22 infections in the Rhythms cohort at 3‐hourly intervals for 33 h from the ZT18 on the first day following infection (i.e. during 42–75 and 30–63 h post‐infection (HPI) in the aligned and misaligned groups, respectively). The proportion of parasites at each IDC stage was quantified by staging 200 parasites per thin blood smear, stained with 20% Giemsa, based on their morphology (Cambie et al., 1991; Prior et al., 2018). Technical issues precluded staging for the ZT12 sample, and where parasitaemia was very low (<0.2%; 29 out of 264 samples) at least 50 parasites were scored. Microscopic analysis was carried out blinded. We verified that there were no differences in RBC density during days 2 and 3 PI that could confound IDC stage data (Table S2).

### 2.4 Data analysis

To analyse parasite performance (total parasite and gametocyte densities) and disease severity (weight and RBC density) in the Fitness cohort, we quantified parasite dynamics over the course of infections as well as summary metrics comprising peaks or minima and cumulative counts. To calculate cumulative variables, we included only mice with a complete set of 14 daily samples. In total, we excluded five mice that were euthanised when reaching the pre‐determined humane end points of infection (CW‐VIR aligned: *n* = 1, 2 and 1 for days 7, 8 and 12 PI, respectively, and CW‐VIR misaligned: *n* = 1 for day 8 PI). Humane end points consist of physiological metrics and behavioural signs that are exhibited by mice unlikely to recover from infections. All data from all mice were included in analyses of dynamics since patterns are less affected by missing samples. Gametocytes are produced in two distinct waves during infections, so we separately consider densities for the first (day 3–8 PI) and the second (day 9–16 PI) peaks of these waves.

We characterised IDC rhythms from the proportion of asexual parasites at ring stage present in each sample from the Rhythms cohort using R (version 4.1.0; R Foundation for Statistical Computing, Vienna, Austria, https://www.R‐project.org/) and the packages ‘MetaCycle’ (function *meta2d*), which integrates JTK_CYCLE, Lomb‐Scargle and ARSER (Wu et al., 2016) and ‘BiocManager’ (function *rain*; Thaben & Westermark, 2014). The rhythmicity of ring stages was verified with both methods, and only mice with *p* values <0.05 (i.e. where the proportion of ring stages was deemed rhythmic by both functions; *n* = 5 out of 5 per group for CW‐0 and *n* = 4/6 per group for CW‐VIR) were used to estimate period (the amount of time required to complete an IDC), phase (timing of the ring stage peak) and amplitude (the relative strength of the oscillation, as a proxy for synchrony of the IDC) for each infection individually, using MetaCycle.

We used the R ‘stats’ package (function *lm*; for linear models) to investigate the effects of ‘Genotype’ (CW‐0 or CW‐VIR), ‘Alignment’ (IDC aligned or misaligned to host rhythms) and their interaction for single time points and cumulative measures (‘Baseline weight’, ‘Baseline RBC’, ‘Cumulative total parasite density’, ‘Peak gametocyte density’ (during the first and second peak, respectively), ‘Cumulative gametocyte density’, ‘Cumulative weight’, ‘Cumulative RBC’, ‘Amplitude’, ‘Period’, ‘Phase’, ‘Peak parasite density’, ‘Minimum RBC’ and ‘Minimum weight’). We used mixed effect models (‘lme4’ package, function *lmer*) to investigate the dynamics of ‘Total parasite density’, ‘Gametocyte density’, ‘Weight’ and ‘RBC’, accounting for pseudoreplication by including ‘Mouse ID’ as a random effect. To allow for nonlinear dynamics over time, we included ‘Genotype’ (CW‐0 or CW‐VIR), ‘Alignment’ (aligned or misaligned to host rhythms) as well as ‘Day PI’ and all 2‐ and 3‐way interactions as explanatory variables. We log_10_‐transformed variables where necessary (readouts of total parasite and gametocyte density; Table S3), to meet assumptions of homogeneity of variance. We compared and minimised models using maximum likelihood deletion testing, prioritising the lowest AICc (Akaike Information Criterion (corrected) for small sample sizes; ‘MuMIn’ package) when minimising across non‐nested models. Test statistics and associated *p*‐values are reported for significant results, and full statistical results are shown in the Tables S1–S5. Effect sizes are given as fold differences (the quotients of the relevant means and 95% confidence intervals (CI) calculated using the Fieller method) with the associated mean ± standard error (SEM) for each group. The number of hosts that reached the human end‐point of infection was compared by Fisher's exact test.

## 3 RESULTS

### 3.1 Verifying the assumptions of the experimental design

In aligned infections, the two parasite genotypes *Plasmodium chabaudi* CW‐0 and CW‐VIR differed in virulence in the manner expected (Mackinnon & Read, 1999). Hosts infected with CW‐VIR reached a 1.66‐fold higher peak parasite density (*F*_(1,23)_ = 13.45, *p* = 0.001, mean peak parasites/μL ± SEM = 2.22 ± 0.17 × 10^5^ for CW‐VIR and 1.33 ± 0.17 × 10^5^ for CW‐0), experienced 0.71‐fold lower minimum RBC density (*F*_(1,23)_ = 11.34, *p* = 0.003, mean minimum RBC/mL ± SEM = 1.54 ± 0.06 × 10^9^ for aligned CW‐VIR and 2.17 ± 0.20 × 10^9^ for aligned CW‐0) and a borderline trend for 0.93‐fold lower minimum weight during infection (*F*_(1,23)_ = 4.29, *p* = 0.050, mean minimum weight in grams ± SEM = 18.1 ± 0.3 for aligned CW‐0 and 19.4 ± 0.6 for aligned CW‐VIR; Figure S1A–C). Additionally, more CW‐VIR‐infected hosts (*n* = 5 vs. none of CW‐0) reached their humane end point, although this difference was not significant (*p* = 0.059). Perturbing the alignment between parasite IDC schedule and host rhythm was successful for both genotypes because misaligned parasites had not fully rescheduled their IDC to realign to their host's rhythms during sampling of the “Rhythms cohort”. Specifically, the average proportion of ring stages for aligned infections peaks in the dark phase when the host feeds, but in the light phase for misaligned infections, reflecting the feeding phase of donor hosts (Figure S1D).

### 3.2 Parasite performance

**FIGURE 2:**
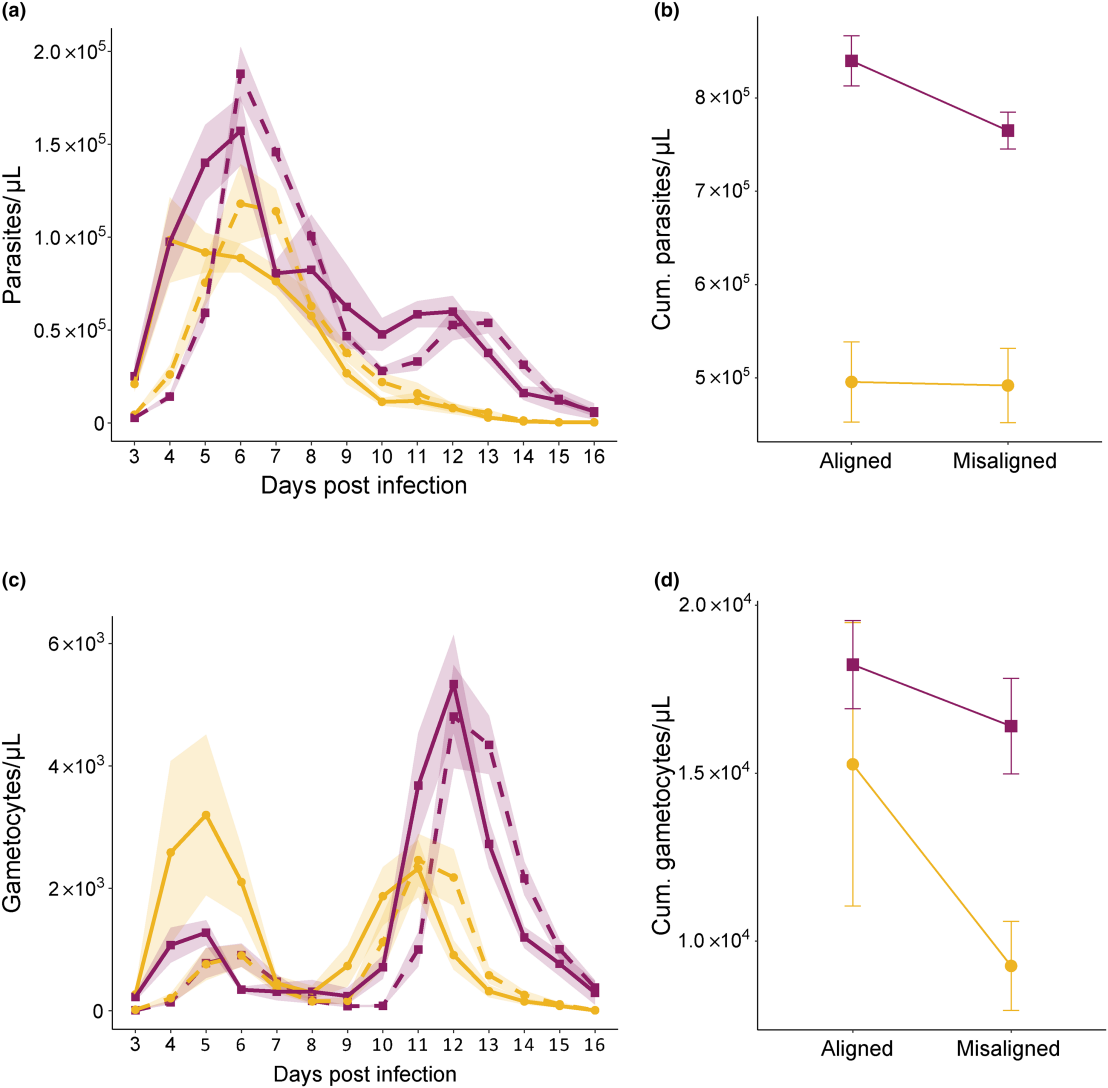
Parasite fitness proxies. The impact of alignment (solid lines) and misalignment (dashed lines) on CW‐0 (yellow circles) and CW‐VIR (pink squares) for measures of parasite performance (mean ± SEM): (a) total parasite density dynamics and (b) cumulative total parasite density, (c) daily gametocyte dynamics and (d) cumulative gametocyte density. Cumulative densities are derived from summing data for individual hosts from days 3 to 16 PI.

We expected CW‐0 to experience a greater fitness cost of misalignment to host rhythms than CW‐VIR. To test this, we first compared parasite densities (as a proxy for within‐host survival), finding that density dynamics differed between genotypes (χ^2^_(13)_ = 82.17, *p* < 0.001) and according to alignment (χ^2^_(13)_ = 278.50, *p* < 0.001; Table S3A; Figure 2a) but not their interaction. Specifically, CW‐VIR exhibited higher densities, including exhibiting a second peak (day 12–13 PI), which CW‐0 did not achieve. Across both genotypes, aligned parasites replicated at higher densities in the first few IDC than in misaligned infections. For example, on day 4 PI, parasite density was 5.04‐fold (95% CI 3.26, 7.69) higher in aligned infections (mean parasites per μL ± SEM = 9.79 ± 1.48 × 10^4^ for aligned and 1.94 ± 0.27 × 10^4^ for misaligned infections). However, across the whole infection, cumulative parasite densities only reflect the difference between genotypes, with CW‐VIR achieving 1.62‐fold (95% CI 1.43, 1.84) higher densities (mean cumulative parasites per μL ± SEM = 7.98 ± 0.18 × 10^5^ for CW‐VIR and 4.94 ± 0.29 × 10^5^ for CW‐0; *F*_(1,43)_ = 68.44, *p* < 0.001; Table S3B; Figure 2b).

Second, we compared gametocyte densities as a proxy for between‐host transmission potential. The temporal dynamics of gametocyte densities differed between the genotypes in manners that depended on their alignment to host rhythms (χ^2^_(13)_ = 35.68, *p* = 0.001; Table S3C; Figure 2c). Specifically, CW‐VIR exhibited a more pronounced second peak in gametocyte densities; aligned infections followed patterns advanced by a day throughout infection; and misalignment affected the magnitude of the early peak for CW‐0 more dramatically. To further explore the impact of alignment and genotype on gametocyte densities, we separately analysed the maximum densities achieved in the first (days 3–8 PI) and second (days 9–16 PI) waves of gametocyte production. Aligned infections produced 2.32‐fold (95% CI 1.08, 4.02) higher peak gametocyte densities during the first wave compared to misaligned infections (mean peak gametocytes per μL ± SEM = 2.57 ± 0.66 × 10^3^ for aligned and 1.11 ± 0.17 × 10^3^ for misaligned infections; *F*_(1,48)_ = 13.55, *p* < 0.001; Table S3D; Figure 2c), irrespective of genotype. However, only genotype explained the densities of the second gametocyte wave, with CW‐VIR achieving 1.94‐fold (95% CI 1.48, 2.61) more gametocytes than CW‐0 (mean peak gametocytes per μL ± SEM = 5.65 ± 0.48 × 10^3^ for CW‐VIR and 2.91 ± 0.32 × 10^3^ for CW‐0; *F*_(1,44)_ = 16.77, *p* < 0.001; Table S3E; Figure 2c). Because the bulk of gametocytes were produced in the second wave, cumulative gametocyte densities across the full duration of infections largely reflected the difference between genotypes, with CW‐VIR achieving 1.40‐fold (95% CI 1.00, 2.25) higher densities (mean cumulative gametocytes per μL ± SEM = 1.72 ± 0.10 × 10^4^ for CW‐VIR and 1.23 ± 0.23 × 10^4^ for CW‐0; *F*_(1,43)_ = 12.94, *p* < 0.001). In addition, we observe a borderline trend (*F*_(1,42)_ = 4.01, *p* = 0.052; Table S3F; Figure 2d) for misalignment to reduce gametocyte density by 1.27‐fold (95% CI 0.88, 1.74), with mean cumulative gametocytes per μL ± SEM = 1.67 ± 0.23 × 10^4^ for aligned and 1.31 ± 0.12 × 10^4^ for misaligned infections.

### 3.3 Disease severity

**FIGURE 3:**
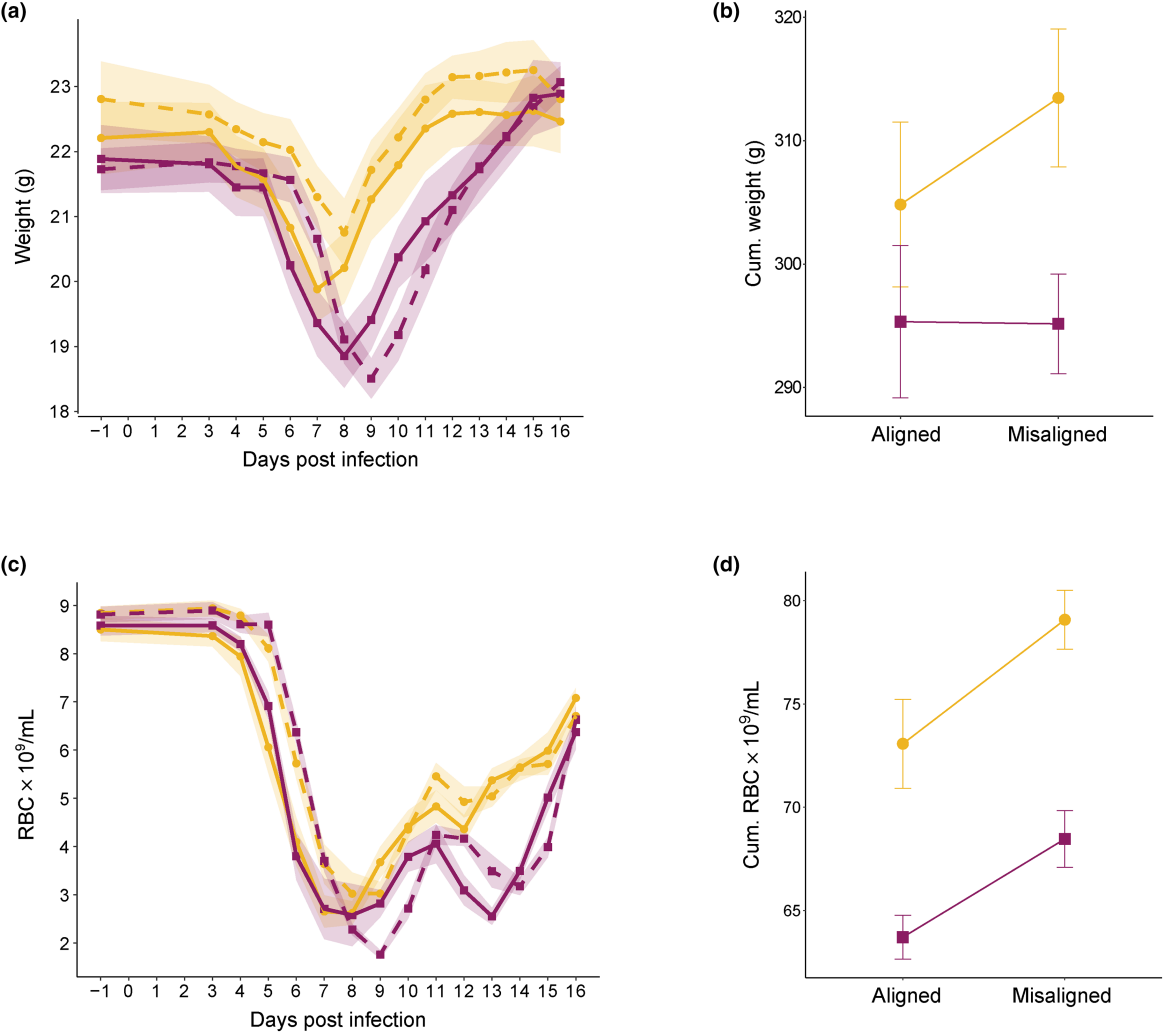
Disease severity proxies. Means ± SEM for aligned (solid lines) and misaligned (dashed lines) parasites of genotypes CW‐0 (yellow circles) and CW‐VIR (pink squares): (a) Mouse weights from days −1 to 16 post‐infection (PI); (b) Cumulative weights, derived from summing data for individual hosts from day 3 to 16 PI; (c) Red blood cell (RBC) density per mL of blood from day −1 to 16 PI; and (d) Cumulative RBCs, derived from summing data for individual hosts from day 3 to 16 PI.

We predicted that misalignment would reduce disease severity and that CW‐0 parasites would be more affected by misalignment than CW‐VIR, and thus, CW‐0 hosts would benefit disproportionately more from their parasites being misaligned. Host weight varied throughout infections, and these dynamics differed for hosts infected with each genotype (χ^2^_(13)_ = 192.16, *p* < 0.001) and according to whether parasites were aligned or misaligned to host rhythms (χ^2^_(13)_ = 55.07, *p* < 0.001; Table S4A; Figure 3a), but not their interaction. As we observed for parasite performance, aligned infections followed dynamics that were advanced by 1 day compared to misaligned infections during the period of weight loss. Hosts infected with CW‐VIR had 1.09‐fold (95% CI 1.04, 1.15) lower minimum weights compared to CW‐0 (mean minimum weight in grams ± SEM = 20.0 ± 0.4 for CW‐0 and 18.3 ± 0.3 for CW‐VIR) and regained their original weight later during recovery (Figure 3a). Over the whole course of infection, any temporal effects of alignment resulted in no net impact on cumulative weight loss, yet the difference between genotypes remained with hosts of CW‐0 retaining 1.05‐fold (95% CI 1.01, 1.09) more weight than those infected with CW‐VIR (mean cumulative weight in grams ± SEM =309.2 ± 4.4 for CW‐0 and 295.2 ± 3.4 for CW‐VIR; *F*_(1,43)_ = 6.35, *p* = 0.016; Table S4B; Figure 3b).

RBC dynamics also varied during infections in manners that depended on whether parasites were aligned or misaligned (χ^2^_(13)_ = 120.68, *p* < 0.001) and on genotype (χ^2^_(13)_ = 122.84, *p* < 0.001), but not their interaction (Table S4C; Figure 3c). Mirroring the dynamics of most other variables, RBC densities of misaligned infections lagged behind those of aligned infections by a day and caused less cumulative anaemia, allowing their hosts to retain 1.07‐fold (95% CI 1.005, 1.14) more RBCs over the course of infections (mean cumulative RBC per mL ± SEM = 73.33 ± 1.47 × 10^9^ for misaligned and 68.62 ± 1.60 × 10^9^ for aligned infections; *F*_(1,42)_ = 11.94, *p* = 0.001; Table S4D; Figure 3d). Hosts infected with CW‐VIR became more anaemic, with 1.38–fold (95% CI 1.16, 1.63) lower minimum RBC densities (mean minimum RBC per mL ± SEM = 2.36 ± 0.13 × 10^9^ for CW‐0 and 1.71 ± 0.11 × 10^9^ for CW‐VIR), experienced two pronounced troughs in RBC density (days 7–9 and 13–14 PI), regaining RBCs later (Figure 3c) and retained 1.15‐fold (95% CI 1.09, 1.20) less RBCs overall during infection (mean cumulative RBC per mL ± SEM = 76.08 ± 1.42 × 10^9^ for CW‐0 and 66.40 ± 1.01 × 10^9^ for CW‐VIR; *F*_(1,42)_ = 41.78, *p* < 0.001; Table S4D; Figure 3d).

Finally, no CW‐0‐infected hosts reached the humane end point of infections, but of the CW‐VIR‐infected hosts, 4 of 14 (29%) with aligned parasites had to be euthanised compared to 1 of 14 (7%) with misaligned infections (*p* = 0.330). Although this difference was not significant, it may suggest a trend towards higher mortality in aligned infections.

### 3.4 Characteristics of the IDC rhythm

**FIGURE 4:**
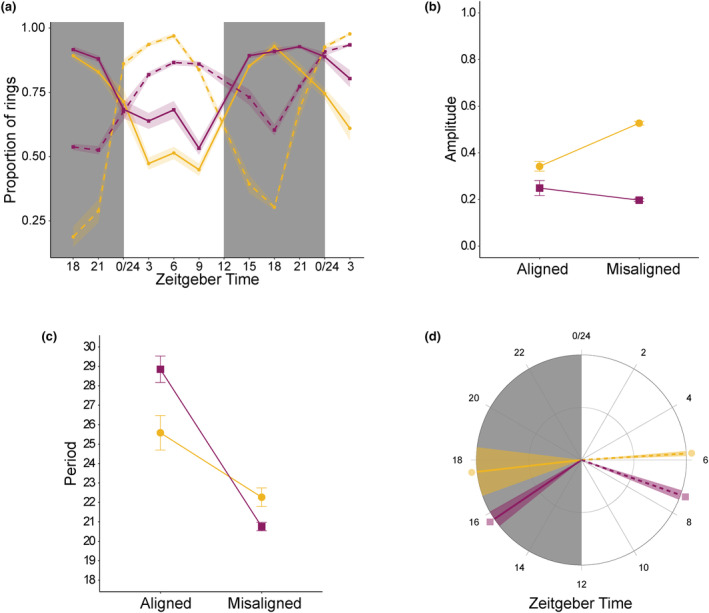
Intraerythrocytic developmental cycle (IDC) rhythms. Means ± SEM for aligned (solid lines) and misaligned (dashed lines) parasites of genotypes CW‐0 (yellow circles) and CW‐VIR (pink squares): (a) proportion of parasites at ring stage, (b) amplitude, (c) period and (d) peak ring phase. Grey shading represents the dark phase of the circadian cycle (i.e. the host's active phase).

We expected that CW‐VIR would be less synchronous than CW‐0 in aligned infections and that CW‐VIR would reschedule faster than CW‐0 following misalignment. According to both functions used, the oscillation of ring stages (Figure 4a) was rhythmic in all CW‐0 infections but only in 8 of 12 CW‐VIR infections (Figure S2), suggesting CW‐VIR is synchronous less often. Moreover, within the rhythmic infections, CW‐VIR had a lower amplitude (i.e. was less synchronous) than CW‐0. This difference between genotypes was more pronounced in misaligned versus aligned infections (*F*_(1,14)_ = 38.64, *p* < 0.001; Table S5A; Figure 4b). Specifically, within aligned infections, the amplitude of ring stage proportions for CW‐VIR parasites was 1.38‐fold (95% CI 1.01, 2.02) lower than for CW‐0 parasites (mean amplitude = 0.25 ± 0.03 for aligned CW‐VIR and 0.34 ± 0.02 for aligned CW‐0), whilst this difference was 2.68‐fold (95% CI 2.47, 2.92) in misaligned infections (mean amplitude = 0.20 ± 0.01 for misaligned CW‐VIR and 0.53 ± 0.01 for misaligned CW‐0).

When misaligned, parasites reschedule by shortening the IDC duration (O'Donnell et al., 2021; Subudhi et al., 2020), which is consistent with a more pronounced reduction in period for CW‐VIR (mean difference = 8.1 h, 95% CI 6.4, 9.8) than CW‐0 (mean difference = 3.3 h, 95% CI 1.0, 5.6; *F*_(1,14)_ = 13.55, *p* = 0.002; Table S5B; Figure 4c). This potential ability of CW‐VIR to reschedule its IDC more effectively is also reflected by the phase estimates for peak ring stage proportion. Specifically, misaligned CW‐VIR parasites were only 8.4 h (95% CI 7.6, 9.3) out of phase to their aligned counterparts, but the phases for aligned and misaligned CW‐0 were 11.8 h (95% CI 9.7, 13.9) apart (*F*_(1,14)_ = 10.06, *p* = 0.007; Table S5C; Figure 4d).

## 4 DISCUSSION

We tested whether virulence correlates with the synchrony of the IDC rhythm by comparing the impact of perturbing the IDC rhythm for two closely genetically related *P. chabaudi* genotypes, which vary in virulence: the relatively avirulent parental CW‐0 and its descendent, the virulent CW‐VIR (Mackinnon & Read, 1999). We predicted that CW‐0 would suffer more than CW‐VIR from the costs of being misaligned, resulting in greater loss of parasite and gametocyte densities, and thereby reducing the severity of infection symptoms. We confirmed that CW‐VIR is more virulent than CW‐0 and found some support for our predictions.

Misaligned infections exhibited approx. 1‐day delayed dynamics for all parasite and host metrics (Figures 2a,c and 3a,c). This may be explained – at least in part – by misaligned infections being initiated 12 h after aligned infections. However, the significant interaction between alignment, genotype and day PI on gametocyte dynamics coupled with lower early gametocyte peaks for both genotypes in misaligned infections (Figure 2c) suggests that in addition to any effect of infection age, misalignment has a direct negative impact on transmission potential. Despite this, the second peak of gametocytes is not affected by alignment, ameliorating the impact of the early peak on overall transmission, especially for CW‐VIR, whose second gametocyte peak is much larger than its first. Misalignment reduced disease severity across infections by slowing weight loss (Figure 3a) and curbing how much RBC hosts lost (Figure 3c,d), but these benefits were not greater for those infected with CW‐0. Furthermore, despite the challenges of estimating absolute values for rhythm parameters from short time series, the qualitative patterns reveal that CW‐VIR is less synchronous, the amplitude of its IDC rhythm is more robust to misalignment, and it shortened its IDC duration more than CW‐0 during rescheduling to bring its phase closer to that of its aligned counterparts (Figure 4). Overall, the impacts of misalignment are transient, becoming eroded as infections progress, likely because parasites reschedule to match the hosts' rhythms (Figure 2b,d). In contrast, the differences between the genotypes are consistent across parasite performance metrics and have greater impact than alignment.

Parasite fitness can be decomposed into traits that underpin host survival and traits facilitating between‐host transmission. Cycles of asexual replication maintain malaria in the host (and gametocytes usually make up less than 1% of parasites (Schneider & Reece, 2021)); thus, we use total parasite density as a proxy for within‐host survival and gametocyte density as an indicator of transmission potential (Cameron et al., 2013). Within‐host survival and between‐host transmission represent a resource allocation trade‐off because the production of asexual stages facilitates within‐host survival via the accumulation of parasite biomass but comes at a short‐term cost of investment into gametocytes, which are required for transmission to mosquitoes (Reece et al., 2009). Indeed, misaligned infections may illustrate this: by producing a lower early gametocyte peak (~57%, mean difference across genotypes = 1.46 × 10^3^ gametocytes per μL, 95% CI 0.10 × 10^3^, 2.83 × 10^3^), misaligned parasites' replication is maximised, which could both ensure safety in numbers against within‐host stressors and safeguard a source population from which to invest in gametocytes later on in the infection (Schneider & Reece, 2021; Westwood et al., 2020). Thus, by reducing early gametocyte investment (Figure 2c), parasites may minimise the costs of misalignment and achieve similar cumulative gametocyte densities as their aligned counterparts (Figure 2d) (Greischar et al., 2016; Schneider, Greischar, et al., 2018). However, erosion of early transmission potential incurs a fitness cost for virulent genotypes, which face a higher risk of host mortality (Greischar et al., 2016). Other non‐mutually exclusive hypotheses for the negative impact of misalignment include elevated gametocyte mortality (O'Donnell et al., 2011) and sampling at the trough of the daily rhythm in gametocyte density due to their maturation and death rates (Pigeault et al., 2018; Schneider, Rund, et al., 2018). Future studies could combine high‐temporal resolution data with a within‐host model of the maturation/senescence rates and density dynamics of gametocytes to explore the roles of plasticity in investment and mortality during misalignment and better predict fitness outcomes and epidemiological impacts.

Our readouts of disease severity are interlinked with parasite density dynamics; weight loss and anaemia become more severe as parasite densities increase, and mortality risk is highest following peak parasite density. Despite no clear impact of misalignment on cumulative total parasite density (Figure 2b), hosts with misaligned parasites did experience less severe cumulative anaemia (Figure 3d) and fewer reached humane end points (for CW‐VIR infections), and misalignment altered the temporal patterns of weight change (but not cumulative weight loss; Figure 3a,b). However, contrary to our expectation, hosts of avirulent parasites (CW‐0) did not disproportionately benefit from the misalignment of their parasites. This may be due to misaligned CW‐0 parasites prioritising asexual replication (via reducing investment into early gametocytes), so they do not disproportionately suffer from misalignment. The relatively minor impacts of misalignment exhibited by both genotypes suggest that early infection processes, potentially including time of day of infection (Hevia et al., 2015; O'Donnell et al., 2014; Sengupta et al., 2019; Stone et al., 2012), have long‐lasting effects on RBC dynamics. For example, more RBCs are lost due to immunopathology than malaria parasite replication, and immunogenicity might be altered when infections occur at different times in the host's circadian cycle (Carvalho Cabral et al., 2022) and by undertaking cytoadherence and sequestration behaviours at an unusual time of day (i.e. in the host's rest phase). Occasionally, the effect of misalignment depends on the pathogen's own phase (i.e. IDC stage) at inoculation – for example, starting infections with aligned trophozoites results in less RBC loss compared to misaligned trophozoites (O'Donnell et al., 2014). While parasite phase at the point of inoculation was standardised in our experiment, future research asking how the phases of both parasite and host interact to modulate the effects of misalignment on disease severity in *Plasmodium* as well as other rhythmic pathogens, for example, African trypanosomes (Rijo‐Ferreira et al., 2017), will be informative for knowing whether disrupting rhythms in either party is a useful intervention strategy.

That virulent parasites are less synchronous in aligned infections and do not experience an increase in synchrony when misaligned fits with observations that *P. falciparum* infections in humans, which cause severe pathology, are not always highly synchronous (Dobaño et al., 2007; Simpson et al., 2002; Touré‐Ndouo et al., 2009). However, estimating synchrony in natural infections is very challenging. For example, using stage proportions overestimates synchrony in expanding infections (Greischar et al., 2019). Thus, because CW‐VIR density increases faster than CW‐0, we are likely to have an overestimated synchrony of CW‐VIR. This makes our finding that CW‐VIR is less synchronous, a conservative result. If synchrony causes detrimental crowding (Greischar et al., 2014), this cost must be traded off against the benefit of timing for exploiting rhythmic RBC behaviours and/or nutrients that are rhythmic due to the host's feeding–fasting rhythm (Prior et al., 2021). Parasites could minimise the cost of synchrony by increasing their preferred age range for host RBCs (for example, by infecting reticulocytes as well as normocytes), thus expanding their pool of host cells – and mathematical modelling suggests that virulent parasites can employ this strategy (Antia et al., 2008). Future studies could explore the optimal level of synchrony and how the costs of synchrony trade‐off against the benefits of timing by comparing the consequences of desynchronisation (i.e. initiating infections with equal numbers of each IDC stage) and misalignment on genotypes that differ in virulence. Furthermore, synchrony between parasites could bring intrinsic benefits, for example, enhancing cell–cell communication when parasites are at the same IDC stage. Such benefits are likely small but could be tested by conducting experiments in arrhythmic hosts without canonical circadian clocks, which allow the intrinsic impacts of synchrony to be assessed.

Finding that misaligned infections appear to shorten their IDC duration (Figure 4c) adds to the body of evidence that *P. chabaudi* readjusts its rhythm to match the phase of host rhythms by speeding up development (O'Donnell et al., 2021; Subudhi et al., 2020). That we found this change greater for CW‐VIR – and its IDC schedule moved closer to that of its aligned counterparts (Figure 4d) – supports the hypothesis that virulent parasites are better able to recover from misalignment. This finding is in keeping with virulent genotypes being generally superior at tolerating within‐host stressors, including competition with co‐infecting genotypes and antimalarial drugs (Bell et al., 2006; De Roode et al., 2005; Schneider et al., 2008, 2012). If virulent parasites are better at rescheduling, this is unlikely to be due to positive density dependence because the rescheduling rate is independent of density (O'Donnell et al., 2021). Perhaps virulent parasites can better harness adaptive plasticity to modulate the IDC rhythm, similar to their greater capacity to adjust traits such as the sex ratio of their gametocytes under challenging conditions (Reece et al., 2008). Plasticity in the IDC rhythm may have a genetic basis upon which selection acts, because both the duration (‘period’) and synchrony (‘amplitude’) of the IDC vary between *P. chabaudi* genotypes (Prior, 2017) and different strains of the human‐infective malaria parasite (*P. falciparum*) vary in their period (Smith et al., 2020). IDC rhythms also become altered during the peak of infections when hosts are most symptomatic and their own rhythms become dysregulated (O'Donnell et al., 2021; Prior, 2017; Prior et al., 2019); perhaps an enhanced ability to cope with variation in host rhythms contributes to virulence.

## 5 CONCLUSION

Overall, we find modest support for the hypothesis that virulent parasites are less synchronous and better able to cope with the impacts of misalignment. The effects of misalignment on parasite density may be small because our sampling occurred over the full course of acute infections, yet parasites can realign the IDC rhythm within a few cycles (O'Donnell et al., 2021). Tracking the prevalence of virulent parasite strains and identifying circumstances that favour them is of public health importance. Different IDC stages vary in their sensitivity to antimalarials (Caillard et al., 1992; Cambie et al., 1991; Owolabi et al., 2021; Skinner et al., 1996), and misalignment enhances artemisinin efficacy against trophozoites (Owolabi et al., 2021). Innovative interventions could manipulate parasite rhythms to mould them into their most tolerable form for hosts or to schedule the IDC for maximal efficacy of existing drugs. Our data warn that virulent parasites could be doubly tenacious against such interventions, as these parasites may be inherently less susceptible to conventional antimalarials (Schneider et al., 2008, 2012) and might bounce back faster from perturbations to their IDC rhythm than less virulent genotypes. Establishing whether this is the case requires quantifying genetic variation for IDC rhythm parameters in natural *P. falciparum* infections and using in vitro approaches to examine whether virulence‐related traits correlate with plasticity in the IDC schedule.

## CONFLICT OF INTEREST STATEMENT

The authors declare no conflicts of interest.

## Supporting information


Figure S1.

Figure S2.

Table S1.
Table S2.
Table S3.
Table S4.
Table S5.

## Data Availability

Data for this study are available at the Edinburgh DataShare Repository (https://doi.org/10.7488/ds/7722).

## References

[eva13696-bib-0001] Antia, R. , Yates, A. , & de Roode, J. C. (2008). The dynamics of acute malaria infections. I. Effect of the parasite's red blood cell preference. Proceedings of the Royal Society B: Biological Sciences, 275(1641), 1449–1458. 10.1098/rspb.2008.0198 PMC2602713 18364317

[eva13696-bib-0002] Bell, A. S. , de Roode, J. C. , Sim, D. , & Read, A. F. (2006). Within‐host competition in genetically diverse malaria infections: Parasite virulence and competitive success. Evolution, 60(7), 1358–1371. 16929653

[eva13696-bib-0003] Birget, P. L. G. , Prior, K. F. , Savill, N. J. , Steer, L. , & Reece, S. E. (2019). Plasticity and genetic variation in traits underpinning asexual replication of the rodent malaria parasite *Plasmodium chabaudi* . Malaria Journal, 18(1), 222. 31262304 10.1186/s12936-019-2857-0 PMC6604315

[eva13696-bib-0004] Birget, P. L. G. , Repton, C. , O'Donnell, A. J. , Schneider, P. , & Reece, S. E. (2017). Phenotypic plasticity in reproductive effort: Malaria parasites respond to resource availability. Proceedings of the Royal Society B: Biological Sciences, 284(1860), 20171229. 10.1098/rspb.2017.1229 PMC5563815 28768894

[eva13696-bib-0005] Boundenga, L. , Ngoubangoye, B. , Ntie, S. , Moukodoum, N. D. , Renaud, F. , Rougeron, V. , & Prugnolle, F. (2019). Rodent malaria in Gabon: Diversity and host range. International Journal for Parasitology. Parasites and Wildlife, 10, 117–124. 31453086 10.1016/j.ijppaw.2019.07.010 PMC6702409

[eva13696-bib-0006] Caillard, V. , Beauté‐Lafitte, A. , Chabaud, A. G. , & Landau, I. (1992). *Plasmodium vinckei petteri*: Identification of the stages sensitive to arteether. Experimental Parasitology, 75(4), 449–456. 1493877 10.1016/0014-4894(92)90258-c

[eva13696-bib-0007] Cambie, G. , Caillard, V. , Beauté‐Lafitte, A. , Ginsburg, H. , Chabaud, A. , & Landau, I. (1991). Chronotherapy of malaria: Identification of drug‐sensitive stage of parasite and timing of drug delivery for improved therapy. Annales de Parasitologie Humaine et Comparée, 66(1), 14–21. 1883151 10.1051/parasite/199166114

[eva13696-bib-0008] Cameron, A. , Reece, S. E. , Drew, D. R. , Haydon, D. T. , & Yates, A. J. (2013). Plasticity in transmission strategies of the malaria parasite, *Plasmodium chabaudi*: Environmental and genetic effects. Evolutionary Applications, 6(2), 365–376. 23467678 10.1111/eva.12005 PMC3586624

[eva13696-bib-0009] Carvalho Cabral, P. , Olivier, M. , & Cermakian, N. (2019). The complex interplay of parasites, their hosts, and circadian clocks. Frontiers in Cellular and Infection Microbiology, 9, 425. 31921702 10.3389/fcimb.2019.00425 PMC6920103

[eva13696-bib-0010] Carvalho Cabral, P. , Tekade, K. , Stegeman, S. K. , Olivier, M. , & Cermakian, N. (2022). The involvement of host circadian clocks in the regulation of the immune response to parasitic infections in mammals. Parasite Immunology, 44(3), e12903. 34964129 10.1111/pim.12903

[eva13696-bib-0011] Ciuffreda, L. , Zoiku, F. K. , Quashie, N. B. , & Ranford‐Cartwright, L. C. (2020). Estimation of parasite age and synchrony status in *Plasmodium falciparum* infections. Scientific Reports, 10(1), 10925. 32616767 10.1038/s41598-020-67817-6 PMC7331735

[eva13696-bib-0012] Cockburn, I. A. , Mackinnon, M. J. , O'Donnell, A. J. , Allen, S. J. , Moulds, J. M. , Baisor, M. , Bockarie, M. , Reeder, J. C. , & Rowe, J. A. (2004). A human complement receptor 1 polymorphism that reduces *Plasmodium falciparum* rosetting confers protection against severe malaria. Proceedings of the National Academy of Sciences of the United States of America, 101(1), 272–277. 14694201 10.1073/pnas.0305306101 PMC314175

[eva13696-bib-0013] de Niz, M. , Ullrich, A. K. , Heiber, A. , Blancke Soares, A. , Pick, C. , Lyck, R. , Keller, D. , Kaiser, G. , Prado, M. , Flemming, S. , del Portillo, H. , Janse, C. J. , Heussler, V. , & Spielmann, T. (2016). The machinery underlying malaria parasite virulence is conserved between rodent and human malaria parasites. Nature Communications, 7, 11659. 10.1038/ncomms11659 PMC4894950 27225796

[eva13696-bib-0014] de Roode, J. C. , Pansini, R. , Cheesman, S. J. , Helinski, M. E. H. , Huijben, S. , Wargo, A. R. , Bell, A. S. , Chan, B. H. K. , Walliker, D. , & Read, A. F. (2005). Virulence and competitive ability in genetically diverse malaria infections. Proceedings of the National Academy of Sciences of the United States of America, 102(21), 7624–7628. 15894623 10.1073/pnas.0500078102 PMC1140419

[eva13696-bib-0015] Dobaño, C. , Rogerson, S. J. , Taylor, T. E. , McBride, J. S. , & Molyneux, M. E. (2007). Expression of merozoite surface protein markers by *Plasmodium falciparum*‐infected erythrocytes in peripheral blood and tissues of children with fatal malaria. Infection and Immunity, 75(2), 643–652. 17118989 10.1128/IAI.01527-06 PMC1828492

[eva13696-bib-0016] Ferguson, H. M. , MacKinnon, M. J. , Chan, B. H. , & Read, A. F. (2003). Mosquito mortality and the evolution of malaria virulence. Evolution, 57, 2792–2804. 14761058 10.1111/j.0014-3820.2003.tb01521.x

[eva13696-bib-0017] Gautret, P. , Deharo, E. , Tahar, R. , Chabaud, A. G. , & Landau, I. (1995). The adjustment of the schizogonic cycle of *Plasmodium chabaudi chabaudi* in the blood to the circadian rhythm of the host. Parasite, 2(1), 69–74. 9137646 10.1051/parasite/1995021069

[eva13696-bib-0018] Greischar, M. A. , Mideo, N. , Read, A. F. , & Bjørnstad, O. N. (2016). Predicting optimal transmission investment in malaria parasites. Evolution, 70(7), 1542–1558. 27271841 10.1111/evo.12969 PMC4991358

[eva13696-bib-0019] Greischar, M. A. , Read, A. F. , & Bjørnstad, O. N. (2014). Synchrony in malaria infections: How intensifying within‐host competition can be adaptive. The American Naturalist, 183(2), E36–E49. 10.1086/674357 PMC4334120 24464205

[eva13696-bib-0020] Greischar, M. A. , Reece, S. E. , Savill, N. J. , & Mideo, N. (2019). The challenge of quantifying synchrony in malaria parasites. Trends in Parasitology, 35(5), 341–355. 30952484 10.1016/j.pt.2019.03.002

[eva13696-bib-0021] Halberg, F. , Johnson, E. A. , Brown, B. W. , & Bittner, J. J. (1960). Susceptibility rhythm to *E. Coli* endotoxin and bioassay. Proceedings of the Society for Experimental Biology and Medicine, 103, 142–144. 14398944 10.3181/00379727-103-25439

[eva13696-bib-0022] Hawking, F. (1967). The 24‐hour periodicity of microfilariae: Biological mechanisms responsible for its production and control. Proceedings of the Royal Society B: Biological Sciences, 169, 59–76.

[eva13696-bib-0023] Hawking, F. , Worms, M. J. , & Gammage, K. (1968). 24‐ and 48‐hour cycles of malaria parasites in the blood; their purpose, production and control. Transactions of the Royal Society of Tropical Medicine and Hygiene, 62(6), 731–765. 4389153 10.1016/0035-9203(68)90001-1

[eva13696-bib-0024] Heipertz, E. L. , Harper, J. , Lopez, C. A. , Fikrig, E. , Hughes, M. E. , & Walker, W. E. (2018). Circadian rhythms influence the severity of sepsis in mice, via a TLR2‐dependent, leukocyte‐intrinsic mechanism. Journal of Immunology, 201(1), 193–201. 10.4049/jimmunol.1701677 PMC9351006 29760192

[eva13696-bib-0025] Hevia, M. A. , Canessa, P. , Müller‐Esparza, H. , & Larrondo, L. F. (2015). A circadian oscillator in the fungus *Botrytis cinerea* regulates virulence when infecting *Arabidopsis thaliana* . Proceedings of the National Academy of Sciences of the United States of America, 112(28), 8744–8749. 26124115 10.1073/pnas.1508432112 PMC4507220

[eva13696-bib-0026] Hirako, I. C. , Assis, P. A. , Hojo‐Souza, N. S. , Reed, G. , Nakaya, H. , Golenbock, D. T. , Coimbra, R. S. , & Gazzinelli, R. T. (2018). Daily rhythms of TNFα expression and food intake regulate synchrony of *Plasmodium* stages with the host circadian cycle. Cell Host and Microbe, 23(6), 796–808.e6. 29805094 10.1016/j.chom.2018.04.016 PMC6014587

[eva13696-bib-0027] Hunter, F. K. , Butler, T. D. , & Gibbs, J. E. (2022). Circadian rhythms in immunity and host‐parasite interactions. Parasite Immunology, 44(3), e12904. 34971451 10.1111/pim.12904 PMC9285061

[eva13696-bib-0028] Jacobs, R. L. (1964). Role of p‐aminobenzoic acid in *Plasmodium berghei* infection in the mouse. Experimental Parasitology, 15, 213–225. 14191322 10.1016/0014-4894(64)90017-7

[eva13696-bib-0029] Kiessling, S. , Dubeau‐Larameé, G. , Ohm, H. , Labrecque, N. , Olivier, M. , & Cermakian, N. (2017). The circadian clock in immune cells controls the magnitude of *Leishmania* parasite infection. Scientific Reports, 7(1), 10892. 28883509 10.1038/s41598-017-11297-8 PMC5589941

[eva13696-bib-0030] Landau, I. (1965). Description of *Plasmodium chabaudi* n. sp., parasite of African rodents. Comptes Rendus Hebdomadaires Des Séances de l'Académie Des Sciences, 260, 3758–3761. 14339659

[eva13696-bib-0031] Long, G. H. , & Graham, A. L. (2011). Consequences of immunopathology for pathogen virulence evolution and public health: Malaria as a case study. Evolutionary Applications, 4(2), 278–291. 25567973 10.1111/j.1752-4571.2010.00178.x PMC3352548

[eva13696-bib-0032] Mackinnon, M. J. , & Read, A. F. (1999). Selection for high and low virulence in the malaria parasite *Plasmodium chabaudi* . Proceedings of the Royal Society B: Biological Sciences, 266(120), 741–748. 10.1098/rspb.1999.0699 PMC1689830 10331293

[eva13696-bib-0033] Mideo, N. , Reece, S. E. , Smith, A. L. , & Metcalf, C. J. E. (2013). The Cinderella syndrome: Why do malaria‐infected cells burst at midnight? Trends in Parasitology, 29(1), 10–16. 23253515 10.1016/j.pt.2012.10.006 PMC3925801

[eva13696-bib-0034] O'Donnell, A. J. , Greischar, M. A. , & Reece, S. E. (2021). Mistimed malaria parasites re‐synchronize with host feeding‐fasting rhythms by shortening the duration of intra‐erythrocytic development. Parasite Immunology, 44(3), e12898. 34778983 10.1111/pim.12898 PMC9285586

[eva13696-bib-0035] O'Donnell, A. J. , Mideo, N. , & Reece, S. E. (2014). Disrupting rhythms in *Plasmodium chabaudi*: Costs accrue quickly and independently of how infections are initiated. Malaria Journal, 12, 503. 10.1186/1475-2875-12-372 PMC3819465 24160251

[eva13696-bib-0036] O'Donnell, A. J. , Prior, K. F. , & Reece, S. E. (2020). Host circadian clocks do not set the schedule for the within‐host replication of malaria parasites. Proceedings of the Royal Society B: Biological Sciences, 287(1932), 20200347. 10.1098/rspb.2020.0347 PMC7575513 32781954

[eva13696-bib-0037] O'Donnell, A. J. , Schneider, P. , McWatters, H. G. , & Reece, S. E. (2011). Fitness costs of disrupting circadian rhythms in malaria parasites. Proceedings of the Royal Society B: Biological Sciences, 278(1717), 2429–2436. 10.1098/rspb.2010.2457 PMC3125626 21208950

[eva13696-bib-0038] Owolabi, A. T. Y. , Reece, S. E. , & Schneider, P. (2021). Daily rhythms of both host and parasite affect antimalarial drug efficacy. Evolution, Medicine, and Public Health, 9(1), 208–219. 34285807 10.1093/emph/eoab013 PMC8284615

[eva13696-bib-0039] Panda, S. , Hogenesch, J. B. , & Kay, S. A. (2002). Circadian rhythms from flies to human. Nature, 417(6886), 329–335. 12015613 10.1038/417329a

[eva13696-bib-0040] Patke, A. , Young, M. W. , & Axelrod, S. (2020). Molecular mechanisms and physiological importance of circadian rhythms. Nature Reviews. Molecular Cell Biology, 21(2), 67–84. 31768006 10.1038/s41580-019-0179-2

[eva13696-bib-0041] Pichon, G. , & Treuil, J. P. (2004). Genetic determinism of parasitic circadian periodicity and subperiodicity in human lymphatic filariasis. Comptes Rendus Biologies, 327(12), 1087–1094. 15656351 10.1016/j.crvi.2004.09.008

[eva13696-bib-0042] Pigeault, R. , Caudron, Q. , Nicot, A. , Rivero, A. , & Gandon, S. (2018). Timing malaria transmission with mosquito fluctuations. Evolution Letters, 2(4), 378–389. 30283689 10.1002/evl3.61 PMC6122125

[eva13696-bib-0043] Pollitt, L. C. , Mideo, N. , Drew, D. R. , Schneider, P. , Colegrave, N. , & Reece, S. E. (2011). Competition and the evolution of reproductive restraint in malaria parasites. The American Naturalist, 177(3), 358–367. 10.1086/658175 PMC3939351 21460544

[eva13696-bib-0044] Prior, K. F. (2017). The evolutionary ecology of circadian rhythms in malaria parasites. University of Edinburgh.

[eva13696-bib-0045] Prior, K. F. , Middleton, B. , Owolabi, A. T. Y. , Westwood, M. L. , Holland, J. , O'Donnell, A. J. , Blackman, M. J. , Skene, D. J. , & Reece, S. E. (2021). Synchrony between daily rhythms of malaria parasites and hosts is driven by an essential amino acid. Wellcome Open Research, 6, 186. 34805551 10.12688/wellcomeopenres.16894.1 PMC8577053

[eva13696-bib-0046] Prior, K. F. , O'Donnell, A. J. , Rund, S. S. C. , Savill, N. J. , van der Veen, D. R. , & Reece, S. E. (2019). Host circadian rhythms are disrupted during malaria infection in parasite genotype‐specific manners. Scientific Reports, 91, 10905. 10.1038/s41598-019-47191-8 PMC6662749 31358780

[eva13696-bib-0047] Prior, K. F. , Rijo‐Ferreira, F. , Assis, P. A. , Hirako, I. C. , Weaver, D. R. , Gazzinelli, R. T. , & Reece, S. E. (2020). Periodic parasites and daily host rhythms. Cell Host and Microbe, 27(2), 176–187. 32053788 10.1016/j.chom.2020.01.005 PMC7137616

[eva13696-bib-0048] Prior, K. F. , van der Veen, D. R. , O'Donnell, A. J. , Cumnock, K. , Schneider, D. , Pain, A. , Subudhi, A. , Ramaprasad, A. , Rund, S. S. C. , Savill, N. J. , & Reece, S. E. (2018). Timing of host feeding drives rhythms in parasite replication. PLoS Pathogens, 14(2), e1006900. 29481559 10.1371/journal.ppat.1006900 PMC5843352

[eva13696-bib-0049] Reece, S. E. , Drew, D. R. , & Gardner, A. (2008). Sex ratio adjustment and kin discrimination in malaria parasites. Nature, 453(7195), 609–614. 18509435 10.1038/nature06954 PMC3807728

[eva13696-bib-0050] Reece, S. E. , Ramiro, R. S. , & Nussey, D. H. (2009). Plastic parasites: Sophisticated strategies for survival and reproduction? Evolutionary Applications, 2(1), 11–23. 20305703 10.1111/j.1752-4571.2008.00060.x PMC2836026

[eva13696-bib-0051] Rijo‐Ferreira, F. , Pinto‐Neves, D. , Barbosa‐Morais, N. L. , Takahashi, J. S. , & Figueiredo, L. M. (2017). *Trypanosoma brucei* metabolism is under circadian control. Nature Microbiology, 2, 17032. 10.1038/nmicrobiol.2017.32 PMC5398093 28288095

[eva13696-bib-0052] Scheiermann, C. , Kunisaki, Y. , & Frenette, P. S. (2013). Circadian control of the immune system. Nature Reviews. Immunology, 13(3), 190–198. 10.1038/nri3386 PMC4090048 23391992

[eva13696-bib-0053] Schneider, P. , Bell, A. S. , Sim, D. G. , O'Donnell, A. J. , Blanford, S. , Paaijmans, K. P. , Read, A. F. , & Reece, S. E. (2012). Virulence, drug sensitivity and transmission success in the rodent malaria, *Plasmodium chabaudi* . Proceedings of the Royal Society B: Biological Sciences, 279(1747), 4677–4685. 10.1098/rspb.2012.1792 PMC3479731 23015626

[eva13696-bib-0054] Schneider, P. , Chan, B. H. , Reece, S. E. , & Read, A. F. (2008). Does the drug sensitivity of malaria parasites depend on their virulence? Malaria Journal, 7, 257. 19087299 10.1186/1475-2875-7-257 PMC2636820

[eva13696-bib-0055] Schneider, P. , Greischar, M. A. , Birget, P. L. G. , Repton, C. , Mideo, N. , & Reece, S. E. (2018). Adaptive plasticity in the gametocyte conversion rate of malaria parasites. PLoS Pathogens, 14(11), e1007371. 30427935 10.1371/journal.ppat.1007371 PMC6261640

[eva13696-bib-0056] Schneider, P. , & Reece, S. E. (2021). The private life of malaria parasites: Strategies for sexual reproduction. Molecular and Biochemical Parasitology, 244, 111375. 34023299 10.1016/j.molbiopara.2021.111375 PMC8346949

[eva13696-bib-0057] Schneider, P. , Repton, C. , & Reece, S. E. (2019a). DNA extraction from 5μL mouse blood samples (KingFisher Flex 96‐well). 10.17504/protocols.io.86fhzbn

[eva13696-bib-0058] Schneider, P. , Repton, C. , & Reece, S. E. (2019b). RNA extraction from 10μL mouse blood samples (KingFisher flex 96‐well). Protocols.io. 10.17504/protocols.io.88fhztn

[eva13696-bib-0059] Schneider, P. , Rund, S. S. C. , Smith, N. L. , Prior, K. F. , O'Donnell, A. J. , & Reece, S. E. (2018). Adaptive periodicity in the infectivity of malaria gametocytes to mosquitoes. Proceedings of the Royal Society B: Biological Sciences, 285(1888), 20181876. 10.1098/rspb.2018.1876 PMC6191691 30282657

[eva13696-bib-0060] Sengupta, S. , Tang, S. Y. , Devine, J. C. , Anderson, S. T. , Nayak, S. , Zhang, S. L. , Valenzuela, A. , Fisher, D. G. , Grant, G. R. , López, C. B. , & FitzGerald, G. A. (2019). Circadian control of lung inflammation in influenza infection. Nature Communications, 10(1), 4107. 10.1038/s41467-019-11400-9 PMC6739310 31511530

[eva13696-bib-0061] Simpson, J. A. , Aarons, L. , Collins, W. E. , Jeffery, G. M. , & White, N. J. (2002). Population dynamics of untreated *Plasmodium falciparum* malaria within the adult human host during the expansion phase of the infection. Parasitology, 124(3), 247–263. 11922427 10.1017/s0031182001001202

[eva13696-bib-0062] Skinner, T. S. , Manning, L. S. , Johnston, W. A. , & Davis, T. M. E. (1996). *In vitro* stage‐specific sensitivity of *plasmodium falciparum* to quinine and artemisinin drugs. International Journal for Parasitology, 26(5), 519–525. 8818732 10.1016/0020-7519(96)89380-5

[eva13696-bib-0063] Smith, L. M. , Motta, F. C. , Chopra, G. , Moch, J. K. , Nerem, R. R. , Cummins, B. , Roche, K. E. , Kelliher, C. M. , Leman, A. R. , Harer, J. , Gedeon, T. , Waters, N. C. , & Haase, S. B. (2020). An intrinsic oscillator drives the blood stage cycle of the malaria parasite *Plasmodium falciparum* . Science, 368(6492), 754–759. 32409472 10.1126/science.aba4357 PMC7518718

[eva13696-bib-0064] Stone, E. F. , Fulton, B. O. , Ayres, J. S. , Pham, L. N. , Ziauddin, J. , & Shirasu‐Hiza, M. M. (2012). The circadian clock protein timeless regulates phagocytosis of bacteria in *Drosophila* . PLoS Pathogens, 8(1), e1002445. 22253593 10.1371/journal.ppat.1002445 PMC3257305

[eva13696-bib-0065] Subudhi, A. K. , O'Donnell, A. J. , Ramaprasad, A. , Abkallo, H. M. , Kaushik, A. , Ansari, H. R. , Abdel‐Haleem, A. M. , Ben Rached, F. , Kaneko, O. , Culleton, R. , Reece, S. E. , & Pain, A. (2020). Malaria parasites regulate intra‐erythrocytic development duration via serpentine receptor 10 to coordinate with host rhythms. Nature Communications, 11(1), 2763. 10.1038/s41467-020-16593-y PMC7265539 32488076

[eva13696-bib-0066] Thaben, P. F. , & Westermark, P. O. (2014). Detecting rhythms in time series with RAIN. Journal of Biological Rhythms, 29(6), 391–400. 25326247 10.1177/0748730414553029 PMC4266694

[eva13696-bib-0067] Touré‐Ndouo, F. S. , Zang‐Edou, E. S. , Bisvigou, U. , & Mezui‐Me‐Ndong, J. (2009). Relationship between in vivo synchronicity of *Plasmodium falciparum* and allelic diversity. Parasitology International, 58(4), 390–393. 19660576 10.1016/j.parint.2009.07.011

[eva13696-bib-0068] Tuteja, R. (2007). Malaria ‐ an overview. The FEBS Journal, 274(18), 4670–4679. 17824953 10.1111/j.1742-4658.2007.05997.x

[eva13696-bib-0069] Wargo, A. R. , de Roode, J. C. , Huijben, S. , Drew, D. R. , & Read, A. F. (2007). Transmission stage investment of malaria parasites in response to in‐host competition. Proceedings. Biological sciences, 274(1625), 2629–2638. 17711832 10.1098/rspb.2007.0873 PMC1975767

[eva13696-bib-0070] Westwood, M. L. , O'Donnell, A. J. , de Bekker, C. , Lively, C. M. , Zuk, M. , & Reece, S. E. (2019). The evolutionary ecology of circadian rhythms in infection. Nature Ecology and Evolution, 3(4), 552–560. 30886375 10.1038/s41559-019-0831-4 PMC7614806

[eva13696-bib-0071] Westwood, M. L. , O'Donnell, A. J. , Schneider, P. , Albery, G. F. , Prior, K. F. , & Reece, S. E. (2020). Testing possible causes of gameatocyte reduction in temporally out‐of‐synch malaria infections. Malaria Journal, 19(1), 17. 31937300 10.1186/s12936-020-3107-1 PMC6958767

[eva13696-bib-0072] Wu, G. , Anafi, R. C. , Hughes, M. E. , Kornacker, K. , & Hogenesch, J. B. (2016). MetaCycle: An integrated R package to evaluate periodicity in large scale data. Bioinformatics, 32(21), 3351–3353. 27378304 10.1093/bioinformatics/btw405 PMC5079475

