# Supplementary figures and images for: Virulence is associated with daily rhythms in the within‐host replication of the malaria parasite Plasmodium chabaudi
Source: Evol Appl. 2024 May 8;17(5):e13696. doi: 10.1111/eva.13696 (PMC11078297; doi:10.1111/eva.13696)

**A**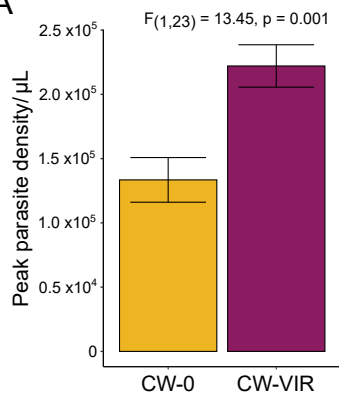**B**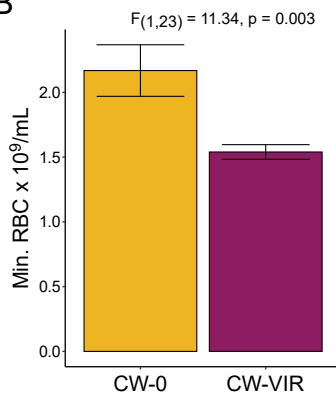**C**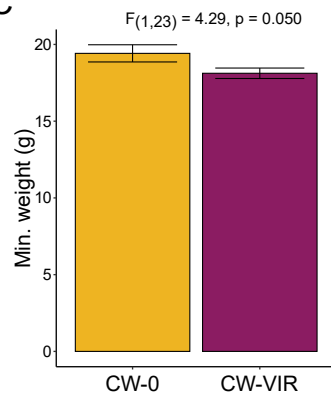**D**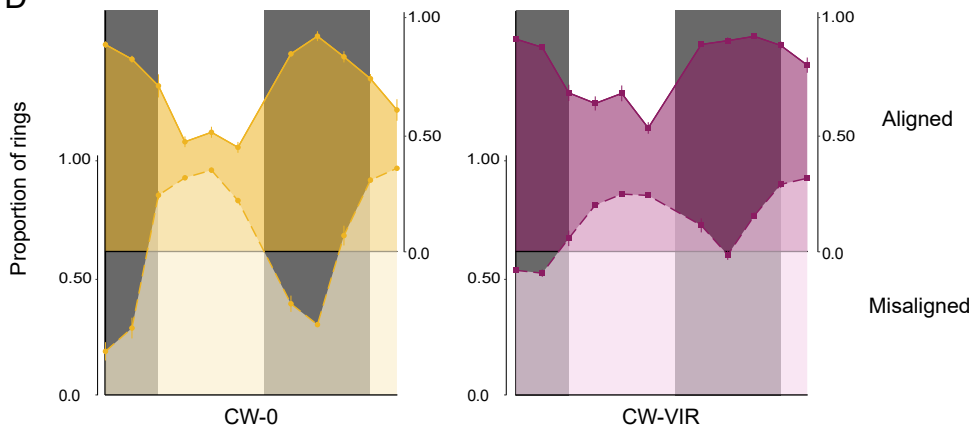

Supplement: Supplementary file 1 — Figure S1. [file EVA-17-e13696-s002.pdf]

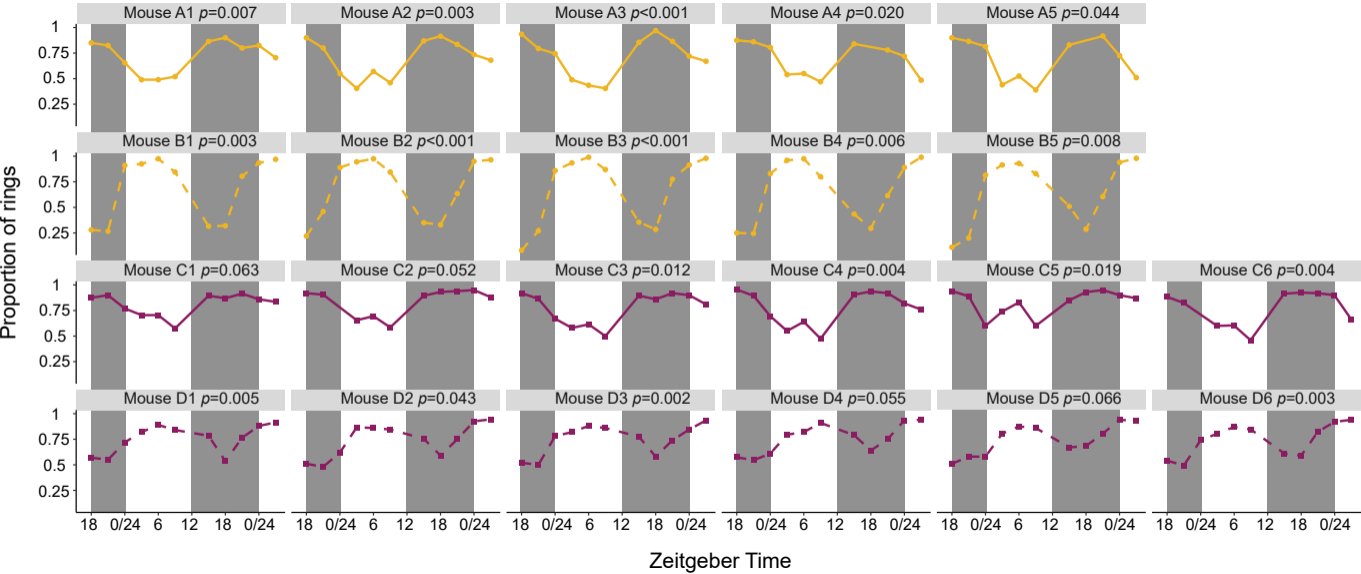

Supplement: Supplementary file 2 — Figure S2. [file EVA-17-e13696-s001.pdf]
