# Supplementary material for: Virulence is associated with daily rhythms in the within‐host replication of the malaria parasite Plasmodium chabaudi
Source: Evol Appl. 2024 May 8;17(5):e13696. doi: 10.1111/eva.13696 (PMC11078297; doi:10.1111/eva.13696)
Supplement: Supplementary file 3 — Table S1. Table S2. Table S3. Table S4. Table S5. [file EVA-17-e13696-s003.docx]

# Supplementary Material

## Supplementary Tables

**Supplementary Table 1. Host health in the Fitness cohort before infection does not differ between treatment groups.**

| *(A) Baseline weight, day -1 PI* | *test statistic* | *p-value* |
| --- | --- | --- |
| Weight~Alignment×Genotype |  |  |
| Alignment×Genotype | *F*_(1,46)_ = 0.58 | *p* = 0.449 |
| Alignment | *F*_(1,47)_ = 0.13 | *p* = 0.721 |
| Genotype | *F*_(1,48)_ = 2.06 | *p* = 0.158 |
| *(B) Baseline RBC, day -1 PI* |  |  |
| RBC~Alignment×Genotype |  |  |
| Alignment×Genotype | *F*_(1,46)_ = 0.08 | *p* = 0.775 |
| Genotype | *F*_(1,47)_ = 0.02 | *p* = 0.898 |
| Alignment | *F*_(1,48)_ = 2.45 | *p* = 0.141 |

Full linear models and results for baseline weight and RBC density one day before infections of the mice used to assess parasite performance and infection severity. Alignment refers to whether the intraerythrocytic developmental cycle (IDC) is aligned or misaligned to host circadian rhythm, and Genotype refers to either the relatively avirulent CW-0 or the more virulent CW-VIR parasites. PI= post infection.

**Supplementary Table 2. RBC density for the Rhythms cohort does not differ between treatment groups during sampling.**

| 1. *RBC of Rhythms cohort – day 2 PI* | *test statistic* | *p-value* |
| --- | --- | --- |
| log_10_(RBC)~Alignment×Genotype |  |  |
| Alignment×Genotype | *F*_(1,18)_ = 3.96 | *p* = 0.062 |
| Alignment | *F*_(1,19)_ = 0.02 | *p* = 0.900 |
| Genotype | *F*_(1,20)_ = 0.39 | *p* = 0.538 |
| *(B) RBC of Rhythms cohort – day 3 PI* |  |  |
| log_10_(RBC)~Alignment×Genotype |  |  |
| Alignment×Genotype | *F*_(1,18)_ = 3.44 | *p* = 0.799 |
| Alignment | *F*_(1,19)_ = 0.28 | *p* = 0.605 |
| Genotype | *F*_(1,20)_ = 0.36 | *p* = 0.557 |

Full linear models and results for RBC density for the days over which the IDC rhythm was characterised. Alignment refers to whether the intraerythrocytic developmental cycle (IDC) is aligned or misaligned to host circadian rhythm, and Genotype refers to either the relatively avirulent CW-0 or the more virulent CW-VIR parasites. PI= post infection.

**Supplementary Table 3. Parasite fitness.**

| *(A) Total parasite density, dynamics days 3-16 PI*  log_10_(Par dens)~Days PI×Alignment×Genotype+(1\|Mouse) | *test statistic* | *p-value* |
| --- | --- | --- |
| Days PI×Alignment×Genotype | χ^2^ = 16.73, df = 13 | *p* = 0.212 |
| Alignment×Genotype | χ^2^ = 0.33, df = 1 | *p* = 0.568 |
| Days PI×Alignment | **χ^2^ = 82.17, df = 13** | ***p* < 0.001** |
| Days PI×Genotype | **χ^2^ = 278.50, df = 13** | ***p* < 0.001** |
| *(B) Cumulative total parasite density days 3-16 PI*  log_10_(Cumulative Par dens)~Alignment×Genotype |  |  |
| Alignment×Genotype | *F*_(1,41)_ = 0.51 | *p* = 0.480 |
| Alignment | *F*_(1,42)_ = 0.62 | *p* = 0.437 |
| Genotype | ***F*_(1,43)_ = 68.44** | ***p* < 0.001** |
| *(C) Gametocyte density, dynamics days 3-16 PI*  log_10_(Gam dens)~Days PI×Alignment×Genotype+(1\|Mouse) |  |  |
| Days PI×Alignment×Genotype | **χ^2^ = 35.68, df = 13** | ***p* = 0.001** |
| *(D) Peak gametocyte density in first peak (days 3-8 PI)*  log_10_(Peak Gam dens)~Alignment×Genotype |  |  |
| Alignment×Genotype | *F*_(1,46)_ = 1.07 | *p* = 0.307 |
| Genotype | *F*_(1,47)_ = 2.08 | *p* = 0.156 |
| Alignment | ***F*_(1,48)_ = 13.55** | ***p* < 0.001** |
| *(E) Peak gametocyte density in second peak (days 9-16 PI)*  log_10_(Peak Gam dens)~Alignment×Genotype |  |  |
| Alignment×Genotype | *F*_(1,42)_ = 0.02 | *p* = 0.899 |
| Alignment | *F*_(1,43)_ = 0.27 | *p* = 0.604 |
| Genotype | ***F*_(1,44)_ = 16.77** | ***p* < 0.001** |
| *(F) Cumulative gametocyte density days 3-16 PI*  log_10_(Cumulative Gam dens)~Alignment×Genotype |  |  |
| Alignment×Genotype | *F*_(1,41)_ = 1.03 | *p* = 0.317 |
| Alignment | *F*_(1,42)_ = 4.01 | *p* = 0.052 |
| Genotype | ***F*_(1,43)_ = 12.94** | ***p* < 0.001** |

Full models and results of linear mixed effects (A, C) and linear (B, D, E, F) models for total parasite and gametocyte densities, with significant terms included in the final models in bold. Alignment refers to whether the IDC is aligned or misaligned to host circadian rhythm, and Genotype refers to either the relatively avirulent CW-0 or the more virulent CW-VIR. PI= post infection. Note: For D and E, all mice contributed to the first gametocyte peak (n = 50) but mice which were euthanised before day 9 PI, were excluded from the analysis of the second gametocyte peak (remaining n = 46).

**Supplementary Table 4.** **Disease severity.**

| *(A) Weight dynamics days 3-16 PI*  Weight~Days PI×Alignment×Genotype+(1\|Mouse) | *test statistic* | *p-value* |
| --- | --- | --- |
| Days PI×Alignment×Genotype | χ^2^ = 12.06, df = 13 | *p* = 0.523 |
| Alignment×Genotype | χ^2^ = 0.28, df = 1 | *p* = 0.599 |
| Days PI×Alignment | **χ^2^ = 55.07, df = 13** | ***p* < 0.001** |
| Days PI×Genotype | **χ^2^ = 192.16, df = 13** | ***p* < 0.001** |
| *(B) Cumulative weight days 3-16 PI*  Cumulative weight~Alignment×Genotype |  |  |
| Alignment×Genotype | *F*_(1,41)_ = 0.62 | *p* = 0.436 |
| Alignment | *F*_(1,42)_ = 0.56 | *p* = 0.458 |
| Genotype | ***F*_(1,43)_ = 6.35** | ***p* = 0.016** |
| *(C) RBC dynamics days 3-16 PI*  RBC~Days PI×Alignment×Genotype+(1\|Mouse) |  |  |
| Days PI×Alignment×Genotype | χ^2^ = 18.67, df = 13 | *p* = 0.134 |
| Alignment×Genotype | χ^2^ = 0.04, df = 1 | *p* = 0.850 |
| Days PI×Alignment | **χ^2^ = 120.68, df = 13** | ***p* < 0.001** |
| Days PI×Genotype | **χ^2^ = 122.84, df = 13** | ***p* < 0.001** |
| *(D) Cumulative RBC days 3-16 PI*  Cumulative RBC~Alignment×Genotype |  |  |
| Alignment×Genotype | *F*_(1,41)_ = 0.16 | *p* = 0.693 |
| Alignment | ***F*_(1,42)_ = 11.94** | ***p* = 0.001** |
| Genotype | ***F*_(1,42)_ = 41.78** | ***p* < 0.001** |

Full models and results of linear mixed effects (A, C) and linear models (B, D) for weight and anaemia, with significant terms remaining in the final models in bold. Alignment refers to whether the IDC is aligned or misaligned to host circadian rhythm, and Genotype refers to either the relatively avirulent CW-0 or the more virulent CW-VIR. PI= post infection. All mice (n = 50) contributed to the dynamics (A, C) but mice (n = 5) which were euthanised during the experiment were excluded from cumulative analyses.

**Supplementary Table 5. Intra-erythrocytic developmental cycle rhythmicity parameters.**

| *(A) Amplitude*  Amplitude~Alignment×Genotype | *test statistic* | *p-value* |
| --- | --- | --- |
| Alignment×Genotype | ***F*_(1,14)_= 38.64** | ***p* < 0.001** |
| *(B) Period*  Period~Alignment×Genotype |  |  |
| Alignment×Genotype | ***F*_(1,14)_= 13.55** | ***p* = 0.002** |
| *(C) Phase*  Phase~Alignment×Genotype |  |  |
| Alignment×Genotype | ***F*_(1,14)_= 10.06** | ***p* = 0.007** |

Full linear models and results for amplitude, period, and phase, with significant terms remaining in the final models in bold. Alignment refers to whether the IDC is aligned or misaligned to host circadian rhythm, and Genotype refers to either the relatively avirulent CW-0 or the more virulent CW-VIR.

## Supplementary Figures


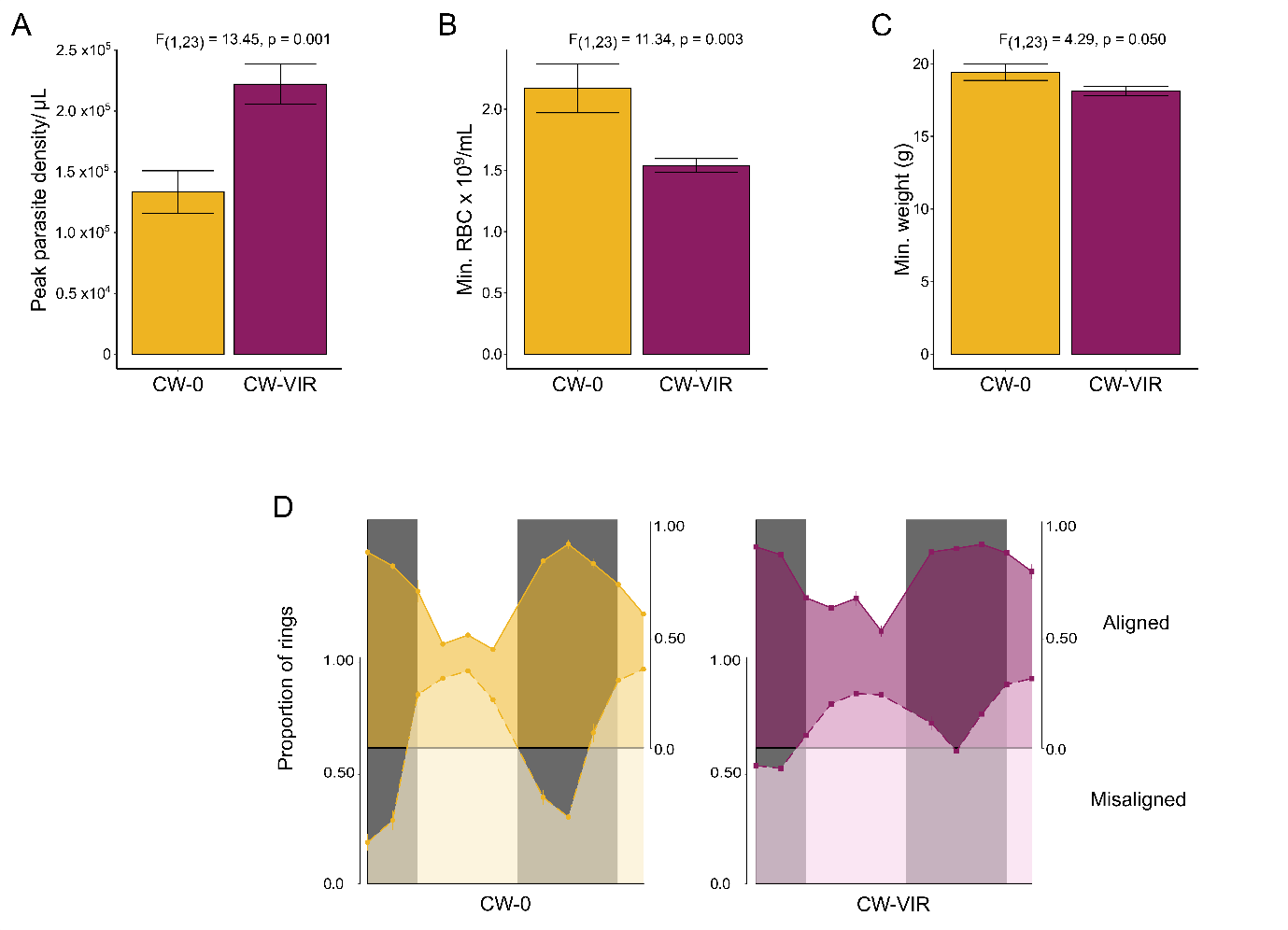


**Supplementary Fig. 1. The assumptions of the experimental design were met.** CW-VIR (N = 14, yellow) is more virulent than CW-0 (N=11, pink) in aligned infections, as shown by (**A**) higher peak parasite densities, (**B**) lower minimum red blood cell density and (**C**) borderline lower minimum weight for CW-VIR compared to CW-0. (**D**) For both CW-VIR (N = 6, pink) and CW-0 (N=5, yellow), ring stage dynamics reveal that this stage peaks in the dark phase in aligned parasites (top graphs with right y-axes, solid lines and dark shading) but in the light phase (bottom graphs with left y-axes, dotted lines and transparent shading) in misaligned parasites, revealing that misalignment was successful and that misaligned parasites have not yet rescheduled. Mean ± S.E.M are shown in all graphs. Grey shading from ZT12-24 indicates lights off (dark phase).

**
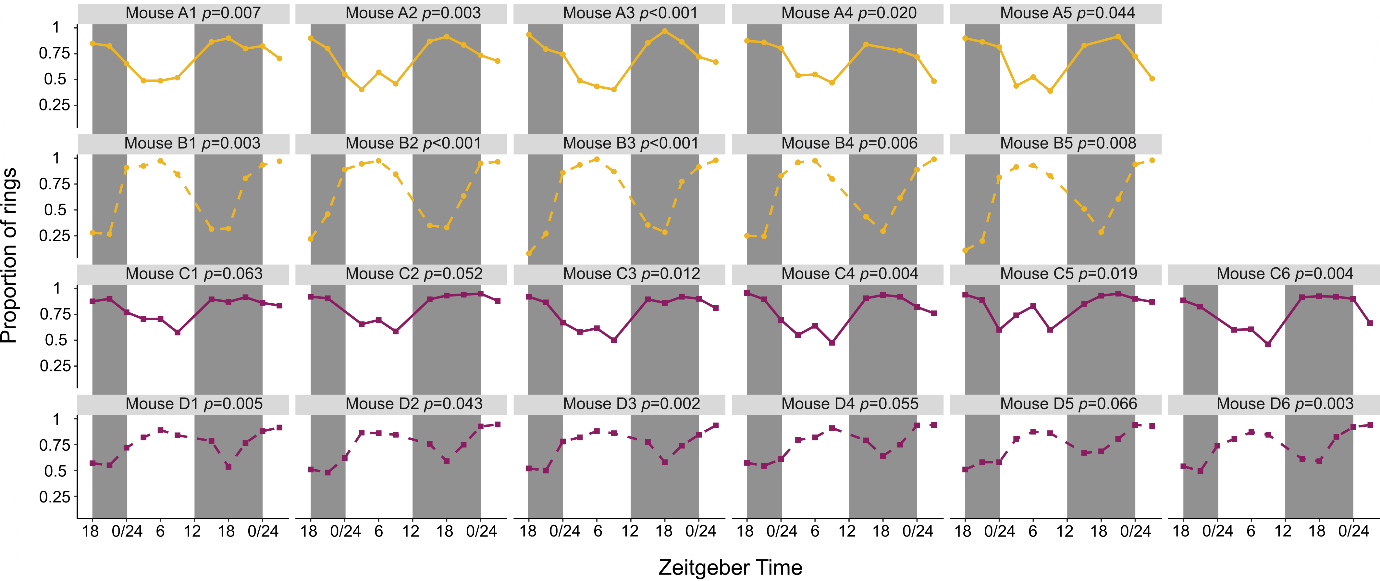
**

**Supplementary Fig. 2. Ring stage rhythm in individual mice.** Proportion of ring stage parasites in the blood from 42-75 hours PI for mice with aligned infections (solid lines) and 30-63 hours PI for mice with misaligned infections (dashed lines), for genotypes CW-0 (yellow) and CW-VIR (pink). Time is presented as Zeitgeber Time (ZT, i.e. hours since lights on). Grey shading from ZT12-24 indicates lights off (dark phase). All mice had rhythmic ring proportions according to the function *rain* (*p* < 0.001) and *p-*values for *meta2d* are indicated in the figure. Only infections deemed significantly rhythmic with both functions were analysed further. Based on these results, mice C1, C2, D4 and D5 have been excluded from analysis for phase, period and amplitude.
